# Mechanical overloading-induced miR-325-3p reduction promoted chondrocyte senescence and exacerbated facet joint degeneration

**DOI:** 10.1186/s13075-023-03037-3

**Published:** 2023-04-04

**Authors:** Jinyun Zhao, Chengjun Li, Tian Qin, Yuxin Jin, Rundong He, Yi Sun, Zhide Liu, Tianding Wu, Chunyue Duan, Yong Cao, Jianzhong Hu

**Affiliations:** 1 Department of Spine Surgery and Orthopaedics, Xiangya Hospital, Central South University, Xiangya Road 87, Changsha, 410008 China; 2 Key Laboratory of Organ Injury, Aging and Regenerative Medicine of Hunan Province, Xiangya Road 87, Changsha, 410008 China; 3 National Clinical Research Center for Geriatric Disorders, Xiangya Hospital, Central South University, Xiangya Road 87, Changsha, 410008 China

**Keywords:** Lumbar facet joints, Degeneration, Mechanical overloading, MiR-325-3p, Chondrocyte senescence

## Abstract

**Objective:**

Lumbar facet joint (LFJ) degeneration is one of the main causes of low back pain (LBP). Mechanical stress leads to the exacerbation of LFJ degeneration, but the underlying mechanism remains unknown. This study was intended to investigate the mechanism of LFJ degeneration induced by mechanical stress.

**Methods:**

Here, mice primary chondrocytes were used to screen for key microRNAs induced by mechanical overloading. SA-β-gal staining, qRT-PCR, western blot, and histochemical staining were applied to detect chondrocyte senescence in vitro and in vivo. We also used a dual-luciferase report assay to examine the targeting relationship of miRNA-325-3p (miR-325-3p) and Trp53. By using NSC-207895, a p53 activator, we investigated whether miR-325-3p down-regulated trp53 expression to reduce chondrocyte senescence. A mice bipedal standing model was performed to induce LFJ osteoarthritis. Adeno-associated virus (AAV) was intraarticularly injected to evaluate the effect of miR-325-3p on facet joint degeneration.

**Results:**

We observed chondrocyte senescence both in human LFJ osteoarthritis tissues and mice LFJ after bipedally standing for 10 weeks. Mechanical overloading could promote chondrocyte senescence and senescence-associated secretory phenotype (SASP) expression. MicroRNA-array analysis identified that miR-325-3p was obviously decreased after mechanical overloading, which was further validated by fluorescence in situ hybridization (FISH) in vivo. Dual-luciferase report assay showed that miR-325-3p directly targeted Trp53 to down-regulated its expression. MiR-325-3p rescued chondrocyte senescence in vitro, however, NSC-207895 reduced this effect by activating the p53/p21 pathway. Intraarticular injection of AAV expressing miR-325-3p decreased chondrocyte senescence and alleviated LFJ degeneration in vivo.

**Conclusion:**

Our findings suggested that mechanical overloading could reduce the expression of miR-325-3p, which in turn activated the p53/p21 pathway to promote chondrocyte senescence and deteriorated LFJ degeneration, which may provide a promising therapeutic strategy for LFJ degeneration.

**Supplementary Information:**

The online version contains supplementary material available at 10.1186/s13075-023-03037-3.

## Introduction

Low back pain (LBP) is a worldwide concern with a lifetime incidence of nearly 80%, which seriously affects the life quality of patients [1, 2]. There are many causes of LBP, such as intervertebral disc degeneration, sacroiliitis, and facet joint syndromes [3, 4]. Facet joint degeneration accounts for about 30% of chronic LBP [4]. Conventional physical therapy does not provide long-term pain relief, and invasive surgical treatments may further damage the articular surface [5, 6]. Therefore, it is particularly significant to seek for potential treatments for facet joint degeneration.

The facet joints and intervertebral disc form a three-joint complex, which is the main loading-bearing structure of the spine [7]. The facet joints are located on both sides of the spine and consist of superior and inferior articular processes. Altered biomechanical structure and disrupted anabolism result in ultimately facet joint degeneration, which was characterized by cartilage thinning, subchondral bone sclerosis, and joint space narrowing [8, 9]. Unlike humans, mice are rodents whose facet joints are basically unaffected by axial forces [10]. Recently, the bipedal standing model has been recognized as an ideal tool for studying facet joint degeneration. After standing for 10 weeks, the facet joints showed obvious degeneration. The Osteoarthritis Research Society International (OARSI) scores and matrix metalloproteinase 13 (MMP13) were increased, while collagen II was decreased [11, 12].

Cellular senescence is a stable state characterized by cell cycle arrest. Senescent cells highly express P53, P21, P16, and senescence-associated-β-galactosidase (SA-β-gal). Senescent cells could release senescence-associated secretory phenotype (SASP) including inflammatory cytokines, chemokines, growth factors, and proteases to affect surrounding cells [13, 14]. SASP factors such as plasminogen activator inhibitor-1 (PAI-1), interleukin-6 (IL-6), and transforming growth factor-β (TGF-β), have been proven to participate in cellular senescence [15, 16]. In joint degeneration, the existence of senescent cells aggravated the destruction of articular cartilage, while inhibition of cellular senescence improved chondrocyte homeostasis and alleviates osteoarthritis progression [17–19]. In knee osteoarthritis, mechanical overloading has been reported to downregulate F-box and WD repeat domain containing 7 (FBXW7) ubiquitin ligase and activate the JNK pathway, therefore promoting chondrocyte senescence [20]. Another research reported that mechanical overloading-induced osteoarthritis was accompanied by the senescence of chondrocytes, while urolithin A could reduce chondrocyte senescence by stabilizing mitochondrial homeostasis, thereby alleviating osteoarthritis progression [21]. However, whether mechanical overloading caused lumbar facet joint chondrocyte senescence and what the specific mechanism was have not yet been reported.

MicroRNAs are a series of non-coding RNAs with a length of 21–23nt, which promote RNA degradation of a targeted gene or inhibit its translation by binding to specific regions of 3′UTR [22]. MicroRNAs are extensively involved in chondrocyte homeostasis and osteoarthritis [23]. Degenerative knee cartilage had low miR-17 expression; administration of exogenous miR-17 reduced the expression of pathological catabolic genes such as metalloproteases and aggrecreptileanases [24]. Another research reported that miR-455 deficiency mice showed pathological manifestations of cartilage degeneration. Intra-articular injection of miR-455 mimics in the destabilization of the medial meniscus (DMM) model suppressed hypoxia-inducible factor-2α (HIF-2α) expression and rescued cartilage degeneration [25]. Facet joint degeneration was also accompanied by microRNA expression alteration. Inhibition of miR-181a-5p using antisense oligonucleotides alleviated the severity of cartilage degeneration of facet joints [26]. Non-coding RNAs including microRNAs play an important role in chondrocyte senescence [27]. MiR-29b-5p has been reported to alleviate chondrocyte senescence with reduced p21 and p16 expression [28]. However, whether mechanical overloading regulated chondrocyte senescence via microRNAs remains unknown.

In the current research, we demonstrated that mechanical overloading-induced miR-325-3p reduction could promote chondrocyte senescence by increasing trp53 expression. Using AAV to upregulate miR-325-3p expression effectively lessened chondrocyte senescence and alleviated the progression of facet joint degeneration, which may provide a potential treatment for facet joint degeneration.

## Methods and materials

### Collection of human lumbar facet joint cartilage samples

Human lumbar FJ OA cartilage specimens were obtained from lumbar disc herniation patients undergoing posterior lumbar interbody fusion (PLIF) surgery at Xiangya Hospital, Central South University. The samples acquired from young lumbar spine facture patients with no history of arthritis disease were applied as normal control. Hematoxylin–eosin (HE) staining, safranin O/fast green, and immunohistochemical analysis were applied to characterize the human lumbar FJ chondrocyte. Before the human lumbar FJ collection, patient consents and the approval of the Ethics Committee of Xiangya Hospital of Central South University (Changsha, China) were obtained (NO: 2020-S415).

### Animals

Two-month-old male C57BL/6 mice were hosted in the animal center at Central South University. Animal studies were approved by the Ethics Committee of Central South University (No: 2021-S121). All animal experimental protocols were in accordance with the National Institutes of Health Guide for the Care and Use of Laboratory Animals.

### Standing LFJ OA model

The procedure of the standing LFJ OA model was followed by the methodology described in a previous literature [11]. Briefly, for the experimental standing model, the mice were placed in a beaker with limited water just above the ankle to induce the bipedal posture. The control group was placed in a similar-sized beaker without adding water. With free access to food and water, these mice were performed the bipedal posture 6 h per day. After 10 weeks of intervention, all mice were anesthetized with 0.3% pentobarbital sodium. Lumbar spines were obtained and fixed in 10% buffered formalin for further experiments.

### Intra-articular delivery of AAV overexpressing miR-325-3p

Adeno-associated virus containing miRNA-325-3p (AAV-miR-325-3p) (Obio Technology (Shanghai)Corp., Ltd., China) was administered 1 day before the start of the standing model protocol. Briefly, after anesthesia by 0.3% pentobarbital sodium, an incision was made on the median dorsal skin at the level of L4/L5. The fascia and muscle tissue were bluntly separated to expose the L4/L5 facet joint capsule. Under a microscope, 1 μl of AAV-miR-325-3p was injected into a bilateral L4/L5 facet joint synovial capsule using a microinjection needle. Then, the muscle and skin were sutured layer by layer, and the mice were returned to their cages after disinfection. Mice were euthanized at 10 weeks post-surgery for lumbar FJ cartilage morphological assessment and histological staining. The mice in the negative control group were treated with the same amount of AAV negative control (AAV-NC).

### Histopathologic and immunohistochemical analysis

The L4–5 lumbar FJs of mice were collected and embedded in paraffin followed by fixation in 4% paraformaldehyde and decalcification in 10% EDTA (PH7.4). Four-micrometer-thick slices of the superior articular joint were obtained and stained with hematoxylin–eosin staining safranin O and fast green (Sigma) to assess lumbar FJ degeneration. OARSI scoring system was applied to characterize the degree of facet joint cartilage degeneration as previously described [29]. For immunohistology, antigen retrieval buffer (Abcam) and blocking agent were used before incubation with corresponding primary antibodies at 4 °C temperatures overnight. Then, the sections were incubated with a secondary antibody for 1 h at room temperature followed by DAB kit, counterstaining with hematoxylin, and mounting to assess the expression of the targeted proteins under the light microscope (Zeiss). All the slides were scored under blinded manner. The antibodies used were listed in Table S1.

### Fluorescence in situ hybridization (FISH)

Constructed biotin-labeled miR-325-3p (Sangon Biotech, China) for fluorescent miRNA in situ hybridization was applied as previously described [30]. Briefly, after decalcification, cryo-cross-sectioned sections were cut into 10-μm thick slides. Then, after dewaxing and rehydrating, the slides were incubated with proteinase K (20 μg/ml, Ambion, USA), followed by prehybridization for 1 h under 37 °C temperature. After removing the prehybridization buffer, the miR-325-3p-biotin probe (5′-UUGAUAGGAGGUGCUCAAUAAA-3′-biotin) was incubated at 60 °C overnight. The following day the slides were incubated with a blocking agent (3% BSA in 0.1% PBST) for 1 h at room temperature after washing with 2 × SSC, 1 × SSC, and 0.5 × SSC, sequentially. Then the slides were incubated with Cy3-conjugated streptavidin (Jackson ImmunoResearch, USA) for 1 h. DAPI was used to label the cell nucleus. Images were captured using an Axio Imager one microscope (Zeiss, Oberkochen, Germany). For quantitative analysis, six independent sections in each group were chosen to evaluate the relative miR-325-3p positive cell ratio using Image J software.

### Mouse primary chondrocyte isolation and culture

The isolation of the mouse primary chondrocyte was performed as previously described [31]. Briefly, three postnatal mice at the age of 5–6 days were anesthetized to death using pentobarbital sodium. Under aseptic conditions, the hindlimb articular cartilage pieces were carefully dissected under microscopic instruments, and incubated twice with collagenase D (3 mg/ml) for 45 min, followed by incubation with collagenase D at a concentration of 0.5 mg/ml overnight. The following day the digested cartilages were centrifugated and resuspended with DMEM/F12 medium (Gibco, USA) containing 10% fetal bovine serum (FBS) and 1% streptomycin/penicillin. The culture medium was changed every 2–3 days, when cells grew at 80–90% confluency, digested with 0.25% trypsin–ethylenediaminetetraacetic acid (trypsin–EDTA) solution, and transferred onto different sizes of dishes according to further experiment requirements. NSC-207895 was used to activate p53 (MedChemExpress, USA). For the in vitro experiments, NSC-207895 was dissolved in dimethyl sulfoxide (DMSO) and added into a chondrocyte culture medium at a concentration of 0.5 μM for 6 h.

### MicroRNA mimic and inhibitor transfection

The miR-325-3p mimic, miR-325-3p inhibitor, and the corresponding negative control (NC) were synthesized by Hanbio Co., China. Lipofectamine 3000 transfection reagent (Thermo Fisher Scientific) was used to transfect microRNA to mouse P1 chondrocyte cells for 48 h based on the manufacturer’s instructions. The cells were then performed with TRIzol (TaKaRa, Tokyo, Japan) to extract RNA or RIPA for immunoblotting analysis. The sequences were listed in Table S2.

### Cyclic tensile strain (CTS) loading of mouse primary chondrocytes

Silicon stretch chambers were applied to seed the mouse primary chondrocytes at a density of 1 × 10 5 cells per chamber after coated with fibronectin. After culturing for 48 h, cyclic tensile strain (CTS) at the parameter of 0.5 Hz, and 5% or 20% elongation was conducted at different duration (0, 6, 12, and 24 h) using a FLEXCELL-5000 mechanical stretch system (Flexcell International, McKeesport, PA, USA) in a CO_2_ incubator. Control cells were defined as the cells seeded into the same chamber and cultured without CTS.

### Senescence associated β-galactosidase (SA-β-gal) staining

SA-β-gal staining was performed by using a SA-β-gal staining kit (Senescence β-Galactosidase Staining Kit #9860, Cell Signaling Technology, USA, MA). Briefly, after removing the culture media and washing with PBS twice, paraformaldehyde-fixed cells were incubated with SA-β-gal working solution at 37 °C overnight. After incubation, the cells were washed with PBS twice and then air-dried. Then the cells were observed using a light microscope (Zeiss, Germany). The SA-β-gal positive cell ratio was counted in three random fields for every culture dish in a blinded manner.

### Immunofluorescence

After primary mice chondrocyte cells were collected at the confluence of 80–90% in cell climbing sheets, the sheets were fixed with PFA and washed with PBS twice. Then the sheets were incubated with 0.3% TBST for 30 min and 1 h blocking buffer (3%BSA in PBS) after washing with PBS twice. After blocking, cells were incubated with primary anti-collagen II antibody and anti-P21 antibody at 4 °C overnight, followed by incubation with the secondary antibody the following day for 1 h. After that, the cells were rinsed with PBS and stained with DAPI to label the cell nucleus. The images of each segment of cells were captured randomly on an Axio Imager one microscope (Zeiss, Oberkochen, Germany). The antibodies used were listed in Table S1.

### Dual-luciferase reporter assay

Dual-luciferase reporter assay was performed to verify the targeting relationship between trp53 and miR-325-3p. The trp53 plasmid and mmu-miR-325-3p mimic were constructed by Hanbio Co., China. Briefly, wild-type 3′UTR of Trp53 containing the mmu-miR-325-3p binding site and the mutated binding sequence was cloned into pSI-Check2 plasmids (Hanbio Co., China), referred to as Trp53-WT and Trp53-MUT, respectively. 3′UTR luciferase reporter constructs (3′UTR-Trp53-wild type, 3′UTR-Trp53-mutant) and miRNAs (miR-325-3p-negative control (NC) or miR-325-3p-mimic) were co-transfected in 293 T cells (a human renal endothelial cell line). After 48 h transfection, the binding relationship between Trp53 and miR-325-3p was detected by applying a Dual-Luciferase Reporter Gene Assay Kit (Beyotime, China) according to the manufacturer’s instructions. A microplate reader was used to measure the luciferase activity of the above-mentioned groups. The sequence of the miR-325-3p mimic was 5′ CUAGACUGAGGCUCCUUGAGG3’. The sequence of miR-325-3p-NC was 5′UCACAACCUCCUAGAAAGAGUAGA3’.

### Western blot

The total protein of cultured chondrocytes (24 h post-microRNA-325-3p mimic or inhibitor intervention) was extracted by using RIPA (Beyotime, China), and the BCA kit was applied to detect the protein concentration. After denaturation, the proteins were fractioned by 10% SDS-PAGE and transferred onto a PVDF membrane (Millipore). Then, the membranes were blocked with 5% fat-free milk and incubated at 4 °C overnight with primary antibodies against p16, p21, p53, and actin. After that, the membranes were washed with 1%TBST twice and incubated with HRP-labeled secondary antibody at room temperature for 1 h. Immunoreactive bands were visualized by using an enhanced chemiluminescence reagent (ECL). Actin was considered as an internal control. The antibodies used were listed in Table S1.

### Quantitative real-time–polymerase chain reaction (qRT-PCR)

The total RNA was extracted from primary chondrocyte cells in the day of 6–7 days post seeding, incubation with microRNA-325-3p mimic or inhibitor for 24 h and corresponding elongation time after CTS by using TRIzol reagent (Invitrogen, USA) and reverse-transcribed to complementary DNA (cDNA) by applying a PrimeScript™ RT Reagent kit (Promega, USA) based on the manufacturer’s instructions. A miRNA First Strand cDNA Synthesis kit (Tailing Reaction, Sangon Biotech, China) was used to perform the reverse transcription of microRNAs. The expression of targeted genes or microRNAs was quantified using a GoTaq qPCR Master Mix kit (Promega, USA) and a quantitative PCR system (ABI, USA). GAPDH was chosen as the internal reference of P16, P21, P53, IL-6, PAI-1, and TGF-β, and U6 was the internal reference of microRNAs. The relative quantitative expression was analyzed using the 2 (− ΔΔCt) method. The following specific primers were listed in Table S3.

### Statistical analysis

GraphPad Prism 8 software was applied to analyze the data. Values of *p* < 0.05 represent statistical significance. All data were presented as the mean ± standard deviations (mean ± SDs). Two-tailed unpaired Student’s t-test and one-way analysis of variance (ANOVA) followed by Tukey’s post hoc tests were used to compare the differences between two groups and multiple groups respectively. All the experiments are designed randomly and the investigators were all blinded to allocation during experiments and evaluation and analysis of the results.

## Results

### Facet joint degeneration was accompanied by cellular senescence in human and mice cartilage

**Fig. 1:**
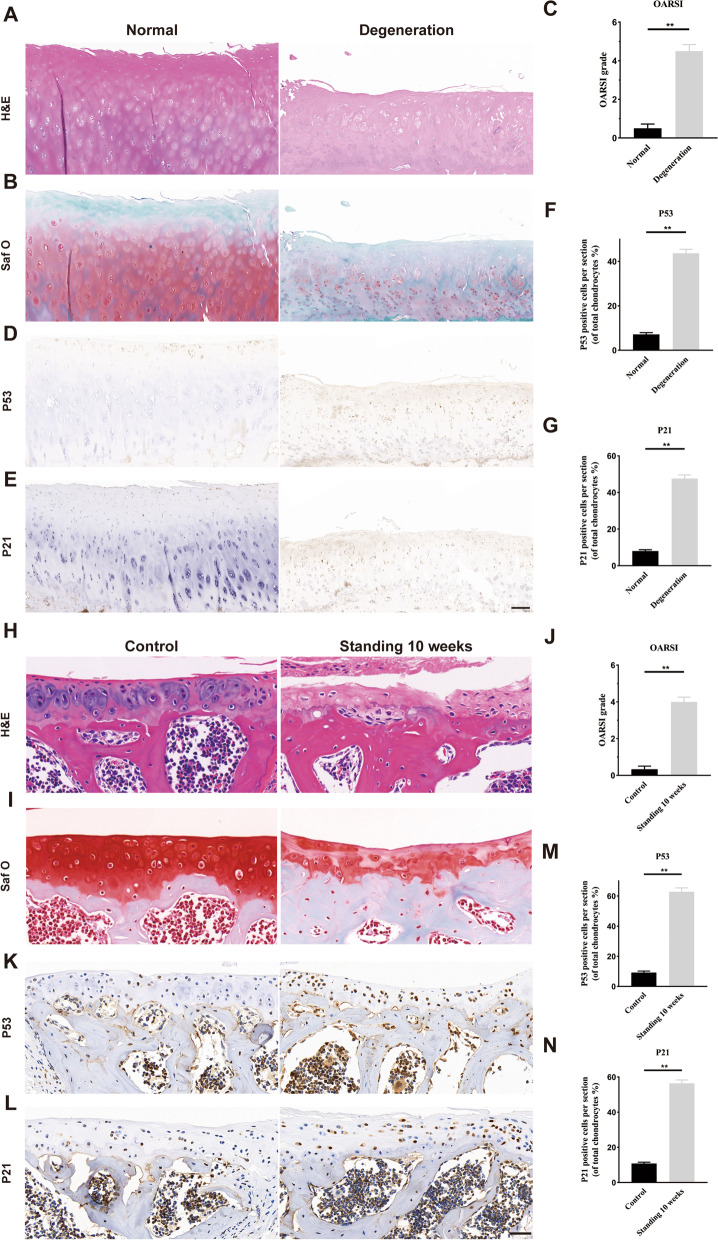
Chondrocyte senescence is associated with lumbar facet joint degeneration in human and mice. **A**, **B** Representative histological images of human LFJ cartilage with hematoxylin–eosin (H&E) and safranin O/fast green in normal and degenerative groups. Scale bar = 200 μm. **C** Quantitative analysis of Osteoarthritis Research Society International (OARSI) score in **B** and **D**, **E** Representative immunohistochemistry images of p53 and p21 in human LFJ cartilage in normal and degenerative groups. Scale bar = 200 μm. **F**, **G** Quantitative analysis of p53 and p21 in **D **and** E**. **H**, **I** Representative histological images of LFJ cartilage with hematoxylin–eosin (H&E) and Safranin O/Fast Green in sham and standing 10 weeks groups in mice. Scale bar = 50 μm. **J** Quantitative analysis of OARSI score in **I**. **K**, **L** Representative immunohistochemistry images of p53 and p21 in LFJ cartilage in sham and standing 10 weeks groups in mice. Scale bar = 50 μm. **M**, **N** Quantitative analysis of p53 and p21 in **K **and **L**. *n* = 6 per group. All data are shown as the mean ± standard deviation (SD). *P < 0.05, **P < 0.01

To explore the impact of degeneration on cartilage senescence, we obtained facet joint cartilages from lumbar disc herniation patients undergoing posterior lumbar interbody fusion (PLIF) surgery and lumbar spine fracture patients with no history of arthritis disease. The HE staining showed that the degenerated FJ exhibited thinning of cartilage and reduced collagen components (Fig. 1A). The safranin O also indicated that the chondrocytes were greatly decreased in degenerated FJs (Fig. 1B). The OARSI scores were increased in degenerated joints, compared to that on normal joints (Fig. 1C). P53 and P21 staining were used to evaluate cell senescence. The P53 positive or P21 positive chondrocytes were increased in degenerated FJ, compared with normal tissues (Fig. 1D–G). We further investigated whether cellular senescence also exists in degenerated FJ in mice, we induced FJ degeneration in mice using a bipedal standing model, which significantly increased spinal axial stress, as previously described [12]. We found that there was no significant difference in the facet joint degeneration of both sides of the standing model mice by HE staining and safranin O staining (Fig. S4). The HE staining and safranin O staining showed that after standing for 10 weeks, the articular cartilage was thinned and chondrocytes were reduced, with increased OARSI scores, when compared to the control group (Fig. 1H–J). Immunohistochemistry (IHC) staining also demonstrated that P53 and P21 positive cells were obviously increased in mice after standing for 10 weeks (Fig. 1K–N). These results suggested that the FJ degeneration exhibited features of osteoarthritis, accompanied by chondrocyte senescence.

### Mechanical overloading-induced chondrocyte senescence and SASP releasing in vitro

**Fig. 2:**
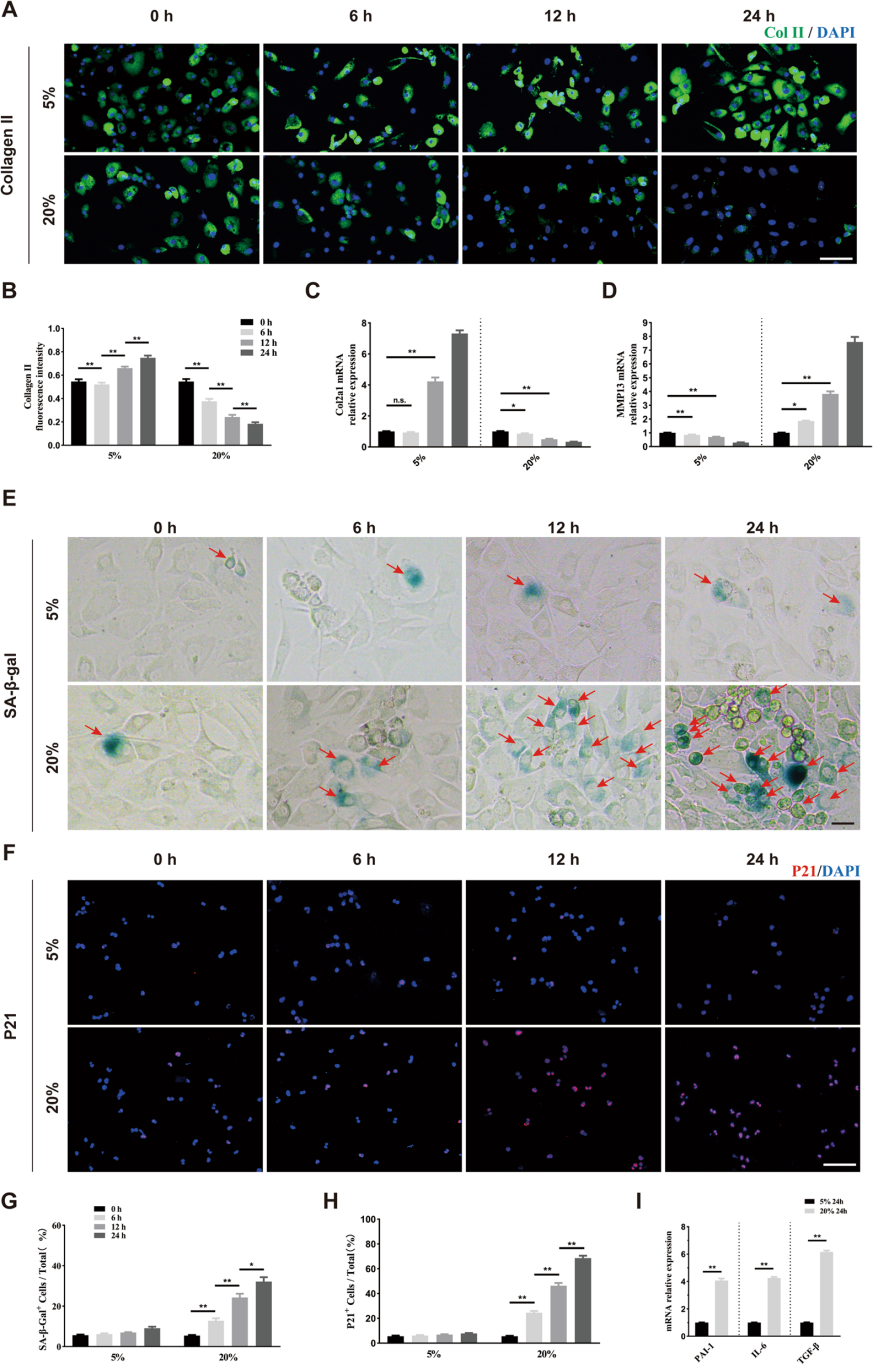
Excessive mechanical loading induces chondrocyte senescence in vitro. **A**, **B** Representative images and quantification of Collagen II staining (green) in primary chondrocytes treated with different elongation strain loading (5% or 20%) for 0, 6, 12, and 24 h. *n* = 6 per time point. Scale bar: 50 μm. **C**, **D** Quantitative PCR analysis of *Col2a1* and *Mmp13* in primary chondrocytes treated with different elongation strain loading (5% or 20%) for 0, 6, 12, and 24 h. *n* = 3 per time point. **E**, **G** Representative images and quantification of SA-β-Gal staining in primary chondrocytes treated with different elongation strain loading (5% or 20%) for 0, 6, 12, and 24 h. Red arrows represent SA-β-Gal.^+^ cells. *n* = 6 per time point. Scale bar: 20 μm. **F**,** H** Representative immunofluorescence images and quantification of P21 (red) in chondrocytes described in **E**. *n* = 6 per time point. Scale bar: 50 μm. **I** Quantitative PCR analysis of SASP-related genes (*PAI-1*, *IL-6*, *TGF-β*) in primary chondrocytes treated with different elongation strain loading (5% or 20%) for 24 h. *n* = 3 per group. All data are shown as the mean ± standard deviation (SD). *P < 0.05, **P < 0.01

To explore whether mechanical loading could affect chondrocyte senescence in vitro, we isolated primary chondrocytes, which were further treated with tensile stress loading of 5% and 20% for 0 h, 6 h, 12 h, and 24 h at a frequency of 0.5 Hz, according to a previous study [20]. The collagen II content showed no significant difference after treating for 6 h, but increased after treating for 12 h and 24 h under 5% tensile stress. Under 20% tensile stress, the collagen II content gradually decreased (Fig. 2A, B). The qRT-PCR of Col2a1 showed a similar tendency (Fig. 2C). As shown in Fig. 2D, the MMP13 expression gradually decreased under 5% tensile stress, while gradually increased under 20% tensile stress in a time-dependent manner. We further stained senescence-associated β-galactosidase (SA-β-gal) and P21 to evaluate mechanical stress on chondrocyte senescence. Under 5% tensile stress, the ratio of SA-β-gal-positive cells showed no statistical difference. When treated with 20% tensile stress, the number of SA-β-gal positive cells increased with prolonged stimulation time (Fig. 2E, G). Immunofluorescence staining of P21 (red) showed a similar trend (Fig. 2F, H). The results of qRT-PCR showed that SASP factors such as *PAI-1*, *IL-6*, and *TGF-β* were greatly elevated under 20% tensile stress for 24 h (Fig. 2I). These results demonstrated that appropriate mechanical stress was beneficial for chondrocyte anabolism, and did not cause chondrocyte senescence. However, excessive mechanical stress was detrimental to cellular anabolism and caused chondrocyte senescence.

### MiR-325-3p was down-regulated in chondrocyte under excessive mechanical stress

**Fig. 3:**
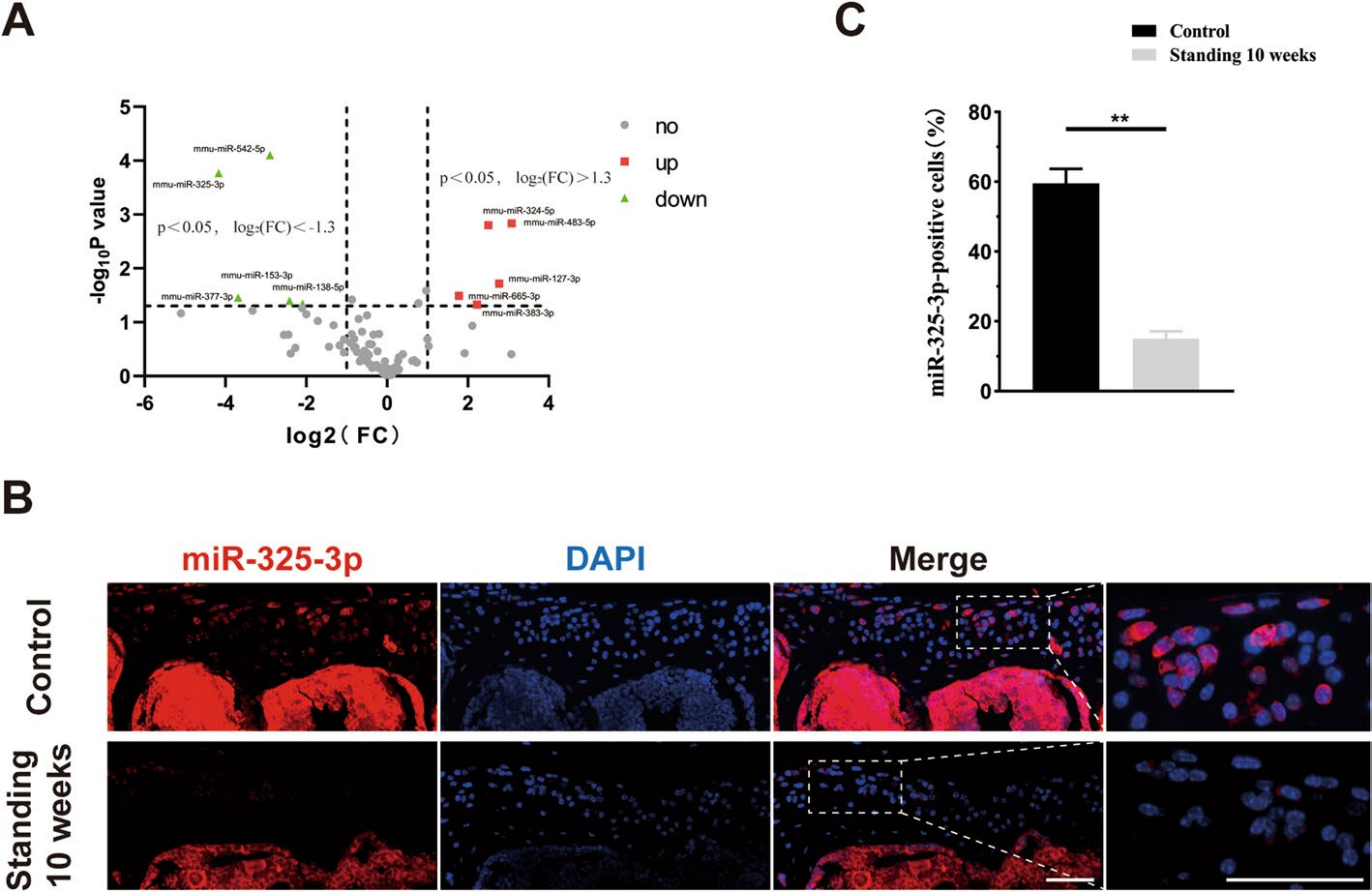
MiR-325-3p was the most down-regulated microRNA in mechanical stress-treated chondrocytes and decreased in standing mice model. **A** Volcano plot of differential microRNAs in mouse chondrocytes with and without mechanical stress (0.5 Hz, 20% elongation, 24 h). **B** In situ hybridization of miR-325-3p (red) and cell nucleus (blue) staining of sham and standing 10 weeks mice. Scale bar = 50 μm. **C** Quantification of the percentage of miR-325-3p positive cells in cartilage area in **B**. *n* = 6 per group. All data are shown as the mean ± standard deviation (SD). **P < 0.01

To explore the mechanism by which mechanical overloading-induced chondrocyte senescence, we performed microRNA-array by selecting joint degeneration and osteoarthritis-related microRNAs. The volcano plot showed that among these microRNAs, miR-325-3p was the most significantly decreased (Fig. 3A). Next, we performed FISH to detection of miR-325-3p expression in lumbar facet joints of mice. As shown in Fig. 3B, C, miR-325-3p was mainly expressed in the cartilage layer and was significantly down-regulated after standing for 10 weeks, compared to the control group. These results indicated that mechanical overloading reduced the expression of miR-325-3p both in vivo and in vitro.

### MiR-325-3p overexpression rescued mechanical overloading-induced chondrocyte senescence in vitro

**Fig. 4:**
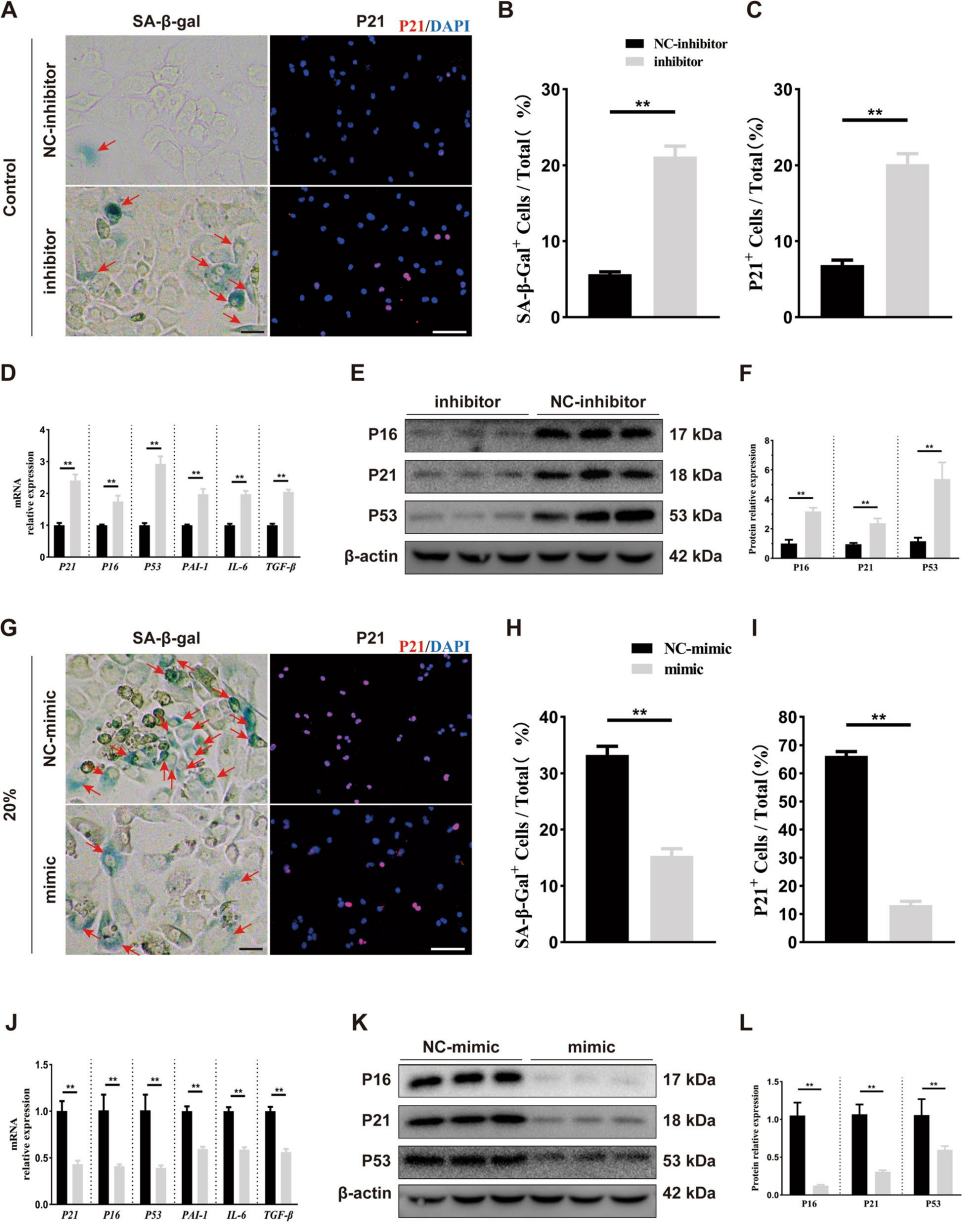
miR-325-3p loss in chondrocytes precipitates senescent states and miR-325-3p overexpression alleviates mechanical stress-induced senescence in vitro. **A** Representative p21 immunofluorescence staining and SA-β-Gal images in chondrocytes treated with miR-325-3p control or inhibitor. Red arrows represent SA-β-Gal^+^ cells. Scale bar: 20 μm in the left SA β-Gal staining panel and 50 μm in the right IF panel. **B**, **C** Quantification of p21 immunofluorescence and SA-β-Gal staining in **A**. *n* = 6 per time point. **D** Quantitative PCR analysis of SASP-related genes (*PAI-1*, *IL-6*, *TGF-β*) in chondrocytes treated with miR-325-3p inhibitor or NC-inhibitor. *n* = 3 per group. **E**, **F** Representative western blot images and quantification in chondrocytes treated with miR-325-3p inhibitor or NC-inhibitor. *n* = 3 per group. **G** Representative p21 immunofluorescence staining and SA-β-Gal images in CTS (20% elongation, 24 h)-treated chondrocytes when administrated with miR-325-3p mimic or NC-mimic. Red arrows represent SA-β-Gal.^+^ cells. Scale bar: 20 μm in the left SA β-Gal staining panel and 50 μm in the right IF panel. **H**,** I** Quantification of p21 immunofluorescence and SA-β-Gal staining in **G**. *n* = 6 per time point. **J** Quantitative PCR analysis of SASP-related genes (*PAI-1*, *IL-6*, *TGF-β*) in CTS (20% elongation, 24 h)-treated chondrocytes treated with miR-325-3p mimic or NC-mimic. *n* = 3 per group. **K**,** L** Representative western blot images and quantification in CTS (20% elongation, 24 h)-treated chondrocytes treated with miR-325-3p mimic or NC-mimic. *n* = 3 per group. All data are shown as the mean ± standard deviation (SD). *P < 0.05, **P < 0.01

To investigate the role of miR-325-3p chondrocyte senescence induced by mechanical overloading, we constructed an miR-325-3p mimic and inhibitor. The results of qRT-PCR verified the transfection efficiency of the mimic and inhibitor (Fig. S1A, S1D). Without tensile stress, the miR-325-3p inhibitor decreased the expression of collagen II (Fig. S1B–C). SA-β-gal staining and P21 (red) staining showed that number of senescent chondrocytes (SA-β-gal and P21 positive cells) was increased after treating with miR-325-3p inhibitor (Fig. 4A–C). The results of qRT-PCR indicated that senescence-related genes *P21*, *P16*, *P53*, and SASP factors *PAI-1*, *IL-6*, and *TGF-β* were up-regulated when treated with miR-325-3p inhibitor (Fig. 4D). Western blot also demonstrated the similar tendency of senescence-related proteins p21, p16, and p53 (Fig. 4E, F). We also administrated miR-325-3p mimic under 20% tensile stress to confirm the effect of miR-325-3p on chondrocyte senescence. As shown in Fig. S1E–F, under mechanical overloading, miR-325-3p mimic administration markedly increased collagen II expression. After treating with 20% tensile stress for 24 h, miR-325-3p mimic could reduce the number of senescent chondrocytes (SA-β-gal positive cells and p21 positive cells) (Fig. 4G–I). The results of qRT-PCR suggested that miR-325-3p mimic obviously lowered expression of senescence-related genes *P21*, *P16*, *P53*, and SASP factors *PAI-1*, *IL-6*, and *TGF-β* (Fig. 4J). Western blot indicated that proteins of p16, p21, and p53 were greatly decreased with miR-325-3p mimic administration under mechanical overloading (Fig. 4K, L). These results suggested that miR-325-3p could rescue mechanical overloading-induced chondrocyte damage and senescence.

### AAV-expressing miR-325-3p attenuated mechanical overloading-induced chondrocyte senescence and FCJ degeneration in a mice bipedal standing model

**Fig. 5:**
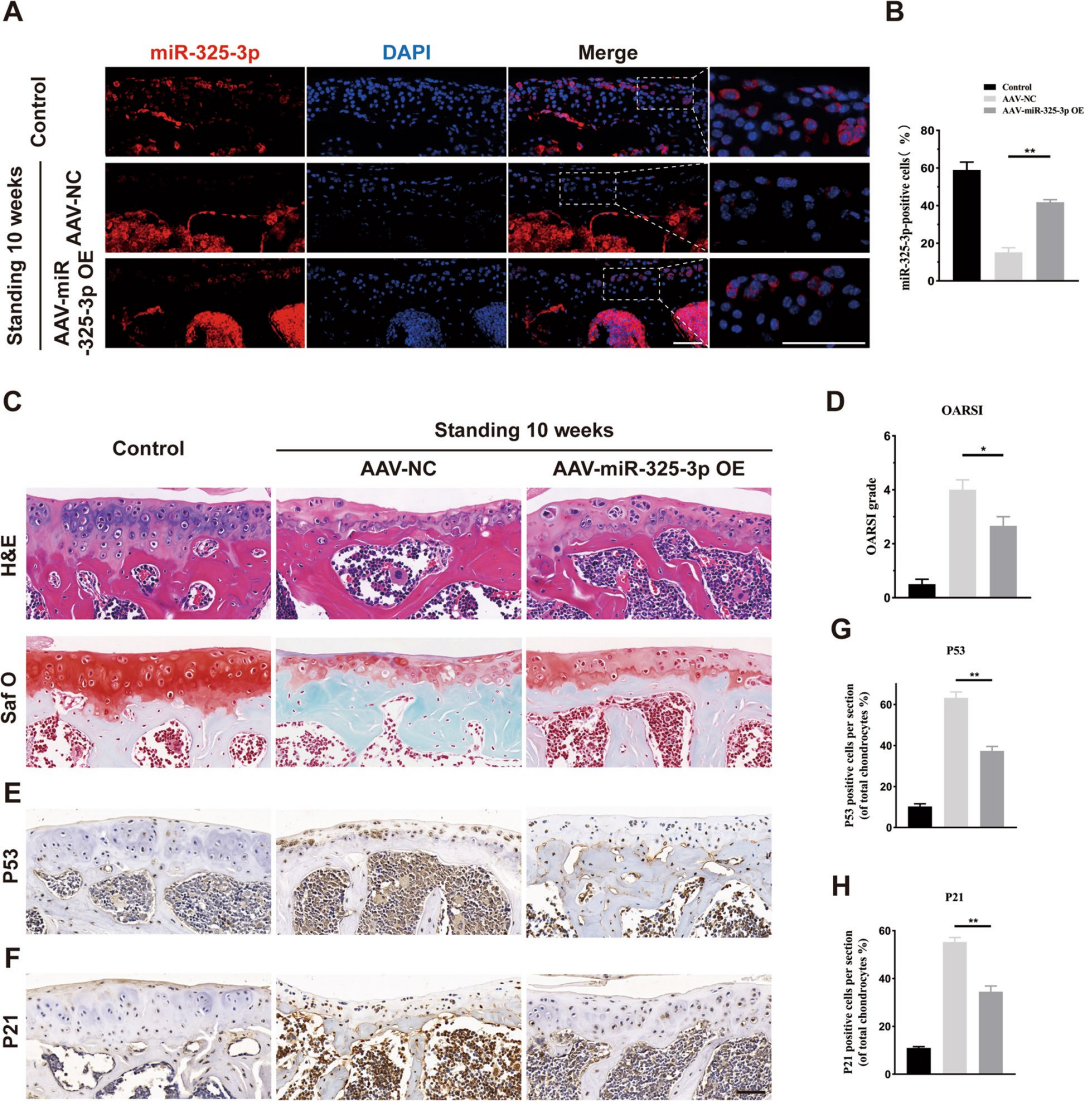
AAV-miR-325-3p OE alleviates lumbar facet joint cartilage senescence in standing model mice. **A** In situ hybridization of miR-325-3p (red) and cell nucleus (blue) staining of sham, AAV-NC-treated groups, and AAV-miR-325-3p OE-treated groups in standing 10 weeks mice. **B** Quantification of the percentage of positive miR-325-3p cells in cartilage area in **A**. **C** Representative histological images of LFJ cartilage with Hematoxylin–eosin (H&E) and Safranin O/Fast Green after 10 weeks standing in sham, AAV-NC-treated groups, and AAV-miR-325-3p OE-treated groups. **D** Quantitative analysis of Osteoarthritis Research Society International (OARSI) score in **C**. **E**, **F** Representative immunohistochemistry images of P53 and P21 in LFJ cartilage in sham, AAV-NC-treated groups, and AAV-miR-325-3p OE-treated groups. **G**, **H** Quantitative analysis of P53 and P21 in **E** and **F**. Scale bar = 50 μm. *n* = 6 per group. All data are shown as the mean ± standard deviation (SD). *P < 0.05, **P < 0.01

To explore the effect of miR-325-3p on chondrocyte senescence and FJ degeneration in vivo, we constructed adeno-associated virus (AAV) expressing miR-325-3p (Obio Technology (Shanghai)Corp., Ltd., China). 1 ul of AAV-miR-325-3p was intra-articular injected into FJ, according to the manufacturer's instructions. To assess the transfection efficiency of AAV-miR-325-3p, we performed FISH experiments. AAV-miR-325-3p promoted the miR-325-3p expression in chondrocytes (Fig. 5A, B). HE and safranin O staining indicated that AAV-miR-325-3p increased cartilage thickness and the number of chondrocytes (Fig. 5C). The OARSI scores were also rose after administration of AAV-miR-325-3p (Fig. 5D). To evaluate the effect of AAV-miR-325-3p on chondrocyte senescence, we performed IHC of p53 and p21. As shown in Fig. 4E–H, compared to the control group, the number of p53 and p21 positive cells were increased in experimental mice receiving AAV-NC, while the senescent chondrocytes were decreased when intra-articular injection of AAV-miR-325-3p. The IHC staining showed that number of collagen II positive and Aggrecan positive chondrocytes were markedly elevated, while MMP 13 positive cells were decreased after AAV-miR-325-3p treatment (Fig. S2). These results indicated that AAV expressing miR-325-3p could effectively reduce chondrocyte senescence and attenuate FJ degeneration induced by mechanical overloading.

### MiR-325-3p reduced chondrocyte senescence by targeting trp53

**Fig. 6:**
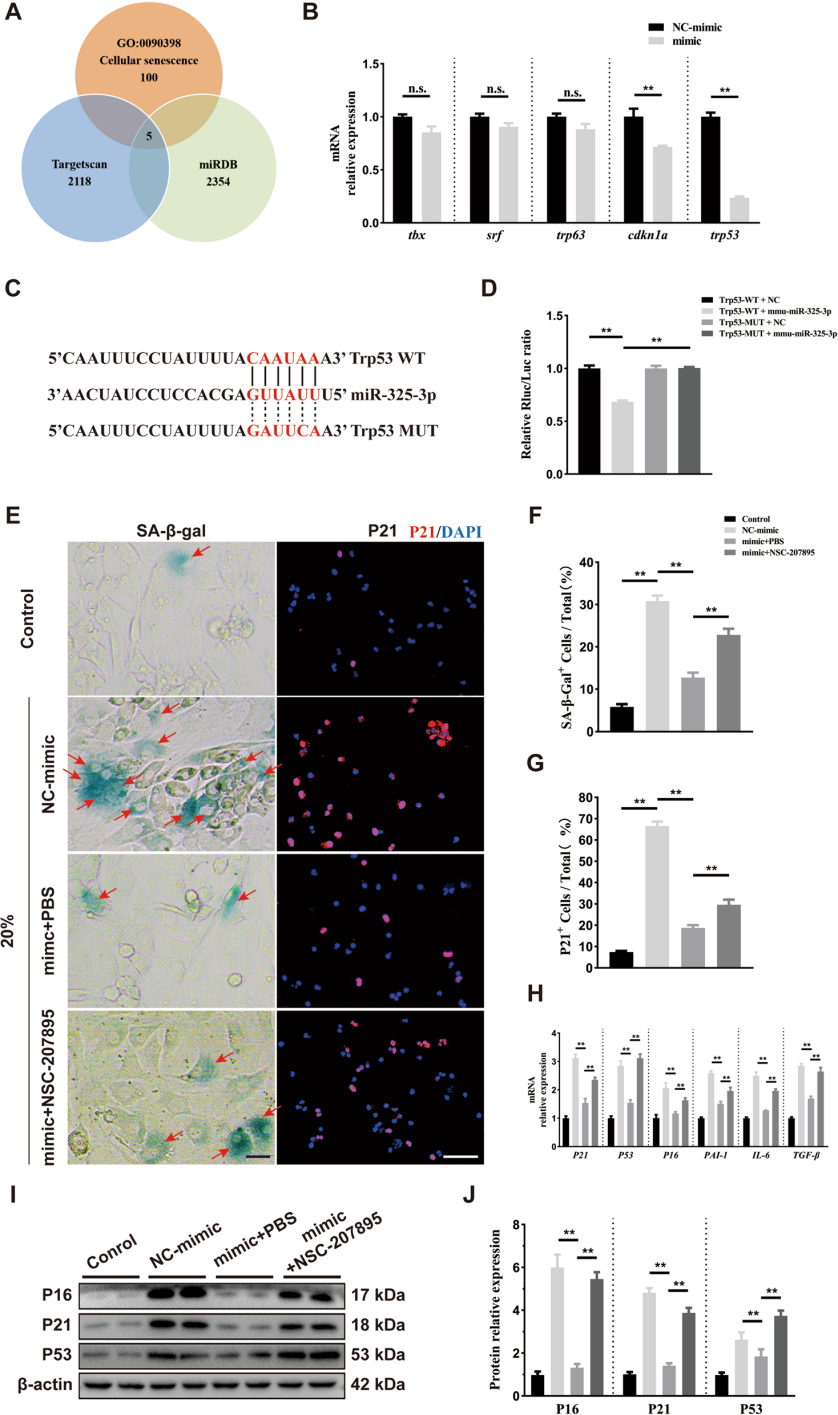
miR-325-3p targeted to P53 and NSC-207895 (P53 activator) reversed miR-325-3p effect on suppressing mechanical stress-induced cellular senescence in vitro. **A** miR-325-3p targeted to multiple genes by TargetScan, miRDB databases, and cellular senescence GO databases (GO:0,090,398). **B** Quantitative PCR analysis of miR-325-3p targeted five genes in mouse chondrocytes treated with miR-325-3p mimic or NC-mimic. *n* = 3 per group. **C** Complementary sequences between miR-325-3p and the 3′ UTR of Trp53. **D** Relative luciferase activities of the p53-wt + negative control group, p53-wt + miR-325-3p group, p53-mut + negative control group, and p53-mut + miR-325-3p group. *n* = 3 per group. **E** Representative p21 immunofluorescence staining and SA-β-Gal images in normal chondrocytes and CTS (20% elongation, 24 h)-treated chondrocytes treated with miR-325-3p control or mimic or mimic plus NSC-207895. Red arrows represent SA-β-Gal.^+^ cells. Scale bar: 20 μm in the left SA β-Gal staining panel and 50 μm in the right IF panel. **F**, **G** Quantification of SA-β-Gal staining and P21 immunofluorescence staining in **E**. *n* = 6 per time point. **H** Quantitative PCR analysis of cellular senescence key genes (*P16*, *P21*, *P53*) and SASP-related genes (*PAI-1*, *IL-6*, *TGF-β*) in chondrocytes described in **E**. *n* = 3 per group. **I**, **J** Representative western blot images and quantification in chondrocytes described in **E**. *n* = 3 per group. All data are shown as the mean ± standard deviation (SD). *P < 0.05, **P < 0.01

We further explored the mechanism by which miR-325-3p regulates chondrocyte senescence. We predicted the downstream target genes of miR-325-3p through Targetscan (targetscan.org/) and miRDB (http://mirdb.org/), and found 5 potential genes (Trp53, TBX, SRF, Trp63, CDKN1A) by intersecting with the aging-related GO pathway (http://geneontology.org/) (Fig. 6A). Subsequently, we found by qRT-PCR that *Trp53* and *CDKN1A* (*P21*) expression in chondrocyte were significantly decreased after treatment with miR-325-3p mimic, while the expression of *TBX*, *SRF*, and *Trp63* showed no difference (Fig. 6B). We used dual-luciferase reporter assay to verify the binding relationship between miR-325-3p and Trp53. As shown in Fig. 6C, the 3′UTR of Trp53 has a sequence complementary to miR-325-3p. The results of the dual-luciferase reporter assay showed that when 293 T cells were transfected with wild-type Trp53 plasmid, miR-325-3p mimic decreased luciferase activity. When 273 T cells were transfected with mutant-Trp53 plasmid, no changes were observed, indicating that miR-325-3p could directly target to Trp53 (Fig. 6D). To further explore whether miR-325-3p suppressed chondrocyte senescence by regulating Trp53, we used a p53 activator, NSC-207895 at a concentration of 0.5 μM. We first explored the effect of NSC-207895 on mechanical-stress chondrocytes after being treated with miR-325-3p mimic. We found that NSC-207895 could reverse the promoted anabolism effect of miR-325-3p on chondrocytes under mechanical overloading (Fig. S3). Then, SA-β-gal staining and P21 (red) staining showed that miR-325-3p decreased mechanical overloading-induced chondrocyte senescence with reduced SA-β-gal positive cells and p21 positive cells, but this effect was attenuated when treating with p53 activator NSC-207895 (Fig. 6E–G). The qRT-PCR and western blot demonstrated that NSC-207895 reduced the effect of miR-325-3p mimic on down-regulating P16, P21, and P53 expression at mRNA and protein levels, respectively (Fig. 6H–J). Meanwhile, miR-325-3p reduced the expression of SASP factors PAI-1, IL-6, and TGF-β; however, NSC-207895 reduced the effect of miR-325-3p to downregulate SASP factors (Fig. 6H). Taken together, these results suggested that miR-325-3p reduced chondrocyte senescence and SASP release by inhibiting Trp53 expression.

## Discussion

Facet joint degeneration is one of the important causes of low back pain [32]. Although mechanical overloading has been reported to promote facet joint degeneration, the specific mechanism remains unknown. In this research, we found that facet joint degeneration was accompanied by chondrocyte senescence, with elevated P53 and P21 expression both in human OA FJ and mice FJ after standing for 10 weeks. MiR-325-3p expression decreased after mechanical overloading using a microRNA array. MiR-325-3p mimic could reduce mechanical overloading-induced collagen II loss, chondrocytes senescence, and SASP release. Local administration of AAV expressing miR-325-3p attenuated facet joint degeneration. Furthermore, trp53 was identified to be a target of miR-325-3p by dual-luciferase reporter assay. The senescence-suppressing effect of miR-325-3p was blocked by the p53 activator, NSC-207895. Our study revealed the specific mechanism of mechanical overloading-induced senescence, which may provide a potential therapeutic approach for facet joint degeneration.

In the process of joint degeneration, pathological changes such as cartilage thickness and collagen reduction, osteophyte formation, and joint space narrowing will occur [33]. In recent years, it has been reported that joint degeneration is accompanied by cartilage senescence, and targeting senescent cells could effectively alleviate the progression of osteoarthritis [34]. The facet joints, also known as zygapophysial joints, together with the intervertebral discs, constitute the force-bearing structure of the spine [5]. The axial force on the spine of rodents is different from that of humans [35]. The bipedal standing model mice can greatly increase the force on the articular surface, simulating the mechanical overload of human facet joints to a certain extent [11, 12]. After standing for 10 weeks, mice showed pathological changes of facet joint degeneration, including thinning of the cartilage layer, disturbance of matrix synthesis, and metabolism [11]. In our research, we also confirmed that excessive mechanical tensile could lead to chondrocyte damage in vitro and in vivo. We further explored the changes of microRNA expression in chondrocytes induced by mechanical stimulation using a microRNA array, indicating that the reduction of miR-325-3p may be a key factor in FJ degeneration caused by mechanical overload.

MicroRNAs are a class of non-coding RNAs with a length of 20–22nt, which can regulate mRNA translation or degradation by binding to the 3′ UTR region [22]. MicroRNAs play an important role in osteoarthritis (OA) and joint degeneration. In knee osteoarthritis, administration of exogenous miR-17 downregulated the expression of pathological catabolic factors such as MMP13, ADAMTS5, and NOS2, thereby reducing extracellular matrix damage and alleviating the severity of osteoarthritis [24]. Nanoparticle-encapsulated miR-141/200c inhibitor could reduce OA progression by modulating SIRT1/IL-6/STAT3 signaling pathway [36]. Another study showed that the exosomes released from hypoxia-preconditioned BMSCs were rich in miR-216a-5p, and these exosomes could promote chondrocyte migration, proliferation, and reduce chondrocyte apoptosis by targeting the JAK2-STAT3 pathway [37]. In FJ degeneration, miR-181a-5p was increased in the chondrocyte. Using antisense oligonucleotides to reduce the expression of miR-181a-5p could inhibit the expression of MMP13 and promote the expression of type II collagen, thereby reducing the progress of FJ degeneration [26]. In our research, 5 up-regulated and 5 down-regulated microRNAs were screened out by microRNA-array, among which the miR-325-3p was the most significantly down-regulated. MiR-325-3p has been shown to contribute to tumor progression and development [38]. Although in the skeletal-muscular system, miR-325-3p had a negative effect on osteoblast differentiation, myogenic differentiation, and osteoclastogenesis; however, whether miR-325-3p could affect chondrocytes has never been reported [39–41]. Our study indicated that chondrocytes treated with mechanical overloading had low expression of miR-325-3p. Use of mimic or AAV to overexpress miR-325-3p could promote the expression of collagen II and reduce the expression of MMP13, thereby attenuating the cartilage damage and matrix destruction caused by mechanical overload.

Cellular senescence is a change characterized by stable cell cycle arrest. Senescent cells highly express senescence markers, such as P53, P21, P16, γ-H2AX, and SA-β-gal, and could also release SASP factors, including metalloenzymes and cytokines, to affect surrounding cells [42, 43]. The process of joint degeneration is also accompanied by the occurrence of cellular senescence. Targeting cellular senescence therapeutic strategies can effectively alleviate osteoarthritis progression [34]. In knee OA, acid-sensitive ion channel 1a promoted the degradation of Lamin B1 protein by inducing autophagy, thereby promoting chondrocyte senescence [44]. Another study showed that the decreased expression of miR-26b-5p during the pathogenesis of OA promoted the expression of Asporin. Asporin further regulated chondrocyte senescence and in destabilization of the medial meniscus (DDM)-induced joint degeneration by inhibiting the TGF-β1/Smad2 signaling pathway [45]. Although it has been confirmed that cartilage aging and OA progression are closely related to knee joint degeneration or osteoarthritis, it is rarely reported whether chondrocyte senescence is related to degeneration progression during facet joint degeneration. It has been reported that the bipedal standing model in mice could increase the expression of p16, a marker of senescence in facet joint chondrocytes [11], but whether microRNAs are involved in regulating chondrocyte senescence is unclear. In our study, we found elevated expression of senescent markers p53 and p21 in human FJ degeneration for the first time. Chondrocyte senescence also occurred in FJ degeneration induced by prolonged bipedal standing in mice, with increased expression of p53 and p21. We confirmed the role of miR-325-3p in regulating chondrocyte senescence and the progression of FJ degeneration both in vitro and in vivo and further verified that miR-325-3p targeted trp53.

There are also limitations to our study. First, we only verified the existence of chondrocyte senescence in human FJ OA, but could not verify the effect of miR-325-3p on chondrocyte senescence in humans FJ OA. Second, we could not rule out whether other differentially-expressed miRNAs affect the chondrocyte senescence. Last, we only observed cartilage changes 10 weeks after standing, and the senescence phenotype of chondrocytes at other time points still needs to be further explored.

## Conclusion

Our study demonstrated that miR-325-3p reduced chondrocyte senescence and alleviated mechanical overloading-induced LFJ degeneration by activating the p53/p21 pathway, which may offer a potential therapeutic strategy for delaying LFJ degeneration progression.

## Supplementary Information

**Additional file 1:** **Figure S1.** The efficiency of miR-325-3p inhibitor and mimic in chondrocytes and the effect of miR-325-3p inhibitor and mimic on Collagen II in vitro. (A) Quantitative PCR analysis of miR-325-3p in mouse chondrocytes treated with miR-325-3p inhibitor or NC-inhibitor. n = 3 per group. (B-C) Representative Collagen II (green) immunofluorescence staining and quantitative analysis in normal chondrocytes treated with miR-325-3p inhibitor or NC-inhibitor. n = 6 per group. Scale bar: 50 µm. (D) Quantitative PCR analysis of miR-325-3p in CTS-treated chondrocytes administrated with miR-325-3p mimic or NC-mimic. n = 3 per group. (E-F) Representative Collagen II (green) immunofluorescence staining and quantitative analysis in CTS-treated chondrocytes administrated with miR-325-3p mimic or NC-mimic. n = 6 per group. Scale bar: 50 µm. All data are shown as the mean ±standard deviation (SD). **P < 0.01. **Figure S2.** AAV-miR-325-3p OE alleviates catabolism and promotes anabolism of chondrocytes in vivo. (A) Representative immunohistochemistry images of Collagen II (top), Aggrecan (middle) and MMP13 (bottom) in LFJ cartilage in sham, AAV-NC-treated groups and AAV-miR-325-3p OE-treated groups after 10 weeks bipedal standing. (B-D) Quantitative analysis of Collagen II, Aggrecan and MMP13 in (A). Scale bar=50 μm. n=6 per group. All data are shown as the mean ±standard deviation (SD). **P < 0.01. **Figure S3.** The effect of NSC-207895 (p53 activator) on mechanical-stress chondrocytes after treated with miR-325-3p mimic. (A) Representative immunofluorescence images of Collagen II in mouse chondrocytes in different groups. (B) Quantitative analysis of Collagen II fluorescence intensity in (A). Scale bar=50 μm. n=6 per group. All data are shown as the mean ±standard deviation (SD). **P < 0.01. **Figure S4.** Symmetrical facet joint degeneration was observed in the bipedal standing mice model. (A-B) Representative histological images of left and right FJs (Facet Joints) with Hematoxylin-eosin (H&E) and Safranin O/Fast Green staining after 10 weeks standing in mice. (C) Quantitative analysis of Osteoarthritis Research Society International (OARSI) score in (B); Scale bar=50 μm. All data are shown as the mean ±standard deviation (SD). n=6 per group. All data are shown as the mean ±standard deviation (SD). ‘n.s.’ represents non-significant (two-tailed Student’s t-test).

**Additional file 2:** **Table S1.** Antibodies. **Table S2.** Sequences of mimic and inhibitor. **Table S3.** Primers for qRT-PCR.

## Data Availability

The data that support the funding of this study are available from the corresponding author upon request. All data relevant to the study are included in the article.

## Abbreviations

LFJ: Lumbar facet joint
LBP: Low back pain
AAV: Adeno-associated virus
SASP: Senescence-associated secretory phenotype
FISH: Fluorescence in situ hybridization
OARSI: Osteoarthritis Research Society International
MMP13: Matrix metalloproteinase 13
SA-β-gal: Senescence-associated-β-galactosidase
PAI-1: Plasminogen activator inhibitor-1
IL-6: Interleukin-6
TGF-β: Transforming growth factor-β
FBXW7: F-box and WD repeat domain containing 7
DMM: Destabilization of the medial meniscus
HIF-2α: Hypoxia-inducible factor-2α
PLIF: Posterior lumbar interbody fusion
HE: Hematoxylin-eosin
AAV-NC: AAV-negative control
FBS: Fetal bovine serum
DMSO: Dimethyl sulfoxide
CTS: Cyclic tensile strain
ECL: Enhanced chemiluminescence reagent
qRT-PCR: Quantitative real-time–polymerase chain reaction
cDNA: Complementary DNA
SDs: Standard deviations
ANOVA: Analysis of variance
IHC: Immunohistochemistry
OA: Osteoarthritis
