# Supplementary material for: Mechanical overloading-induced miR-325-3p reduction promoted chondrocyte senescence and exacerbated facet joint degeneration
Source: Arthritis Res Ther. 2023 Apr 4;25:54. doi: 10.1186/s13075-023-03037-3 (PMC10071751; doi:10.1186/s13075-023-03037-3)
Supplement: Supplementary file 1 — Additional file 1: Figure S1. The efficiency of miR-325-3p inhibitor and mimic in chondrocytes and the effect of miR-325-3p inhibitor and mimic on Collagen II in vitro. (A) Quantitative PCR analysis of miR-325-3p in mouse chondrocytes treated with miR-325-3p inhibitor or NC-inhibitor. n = 3 per group. (B-C) Representative Collagen II (green) immunofluorescence staining and quantitative analysis in normal chondrocytes treated with miR-325-3p inhibitor or NC-inhibitor. n = 6 per group. Scale bar: 50 µm. (D) Quantitative PCR analysis of miR-325-3p in CTS-treated chondrocytes administrated with miR-325-3p mimic or NC-mimic. n = 3 per group. (E-F) Representative Collagen II (green) immunofluorescence staining and quantitative analysis in CTS-treated chondrocytes administrated with miR-325-3p mimic or NC-mimic. n = 6 per group. Scale bar: 50 µm. All data are shown as the mean ±standard deviation (SD). **P < 0.01. Figure S2. AAV-miR-325-3p OE alleviates catabolism and promotes anabolism of chondrocytes in vivo. (A) Representative immunohistochemistry images of Collagen II (top), Aggrecan (middle) and MMP13 (bottom) in LFJ cartilage in sham, AAV-NC-treated groups and AAV-miR-325-3p OE-treated groups after 10 weeks bipedal standing. (B-D) Quantitative analysis of Collagen II, Aggrecan and MMP13 in (A). Scale bar=50 μm. n=6 per group. All data are shown as the mean ±standard deviation (SD). **P < 0.01. Figure S3. The effect of NSC-207895 (p53 activator) on mechanical-stress chondrocytes after treated with miR-325-3p mimic. (A) Representative immunofluorescence images of Collagen II in mouse chondrocytes in different groups. (B) Quantitative analysis of Collagen II fluorescence intensity in (A). Scale bar=50 μm. n=6 per group. All data are shown as the mean ±standard deviation (SD). **P < 0.01. Figure S4. Symmetrical facet joint degeneration was observed in the bipedal standing mice model. (A-B) Representative histological images of left and right FJs (Facet Joints) with Hemat [file 13075_2023_3037_MOESM1_ESM.docx]

**Supplement Figures:**


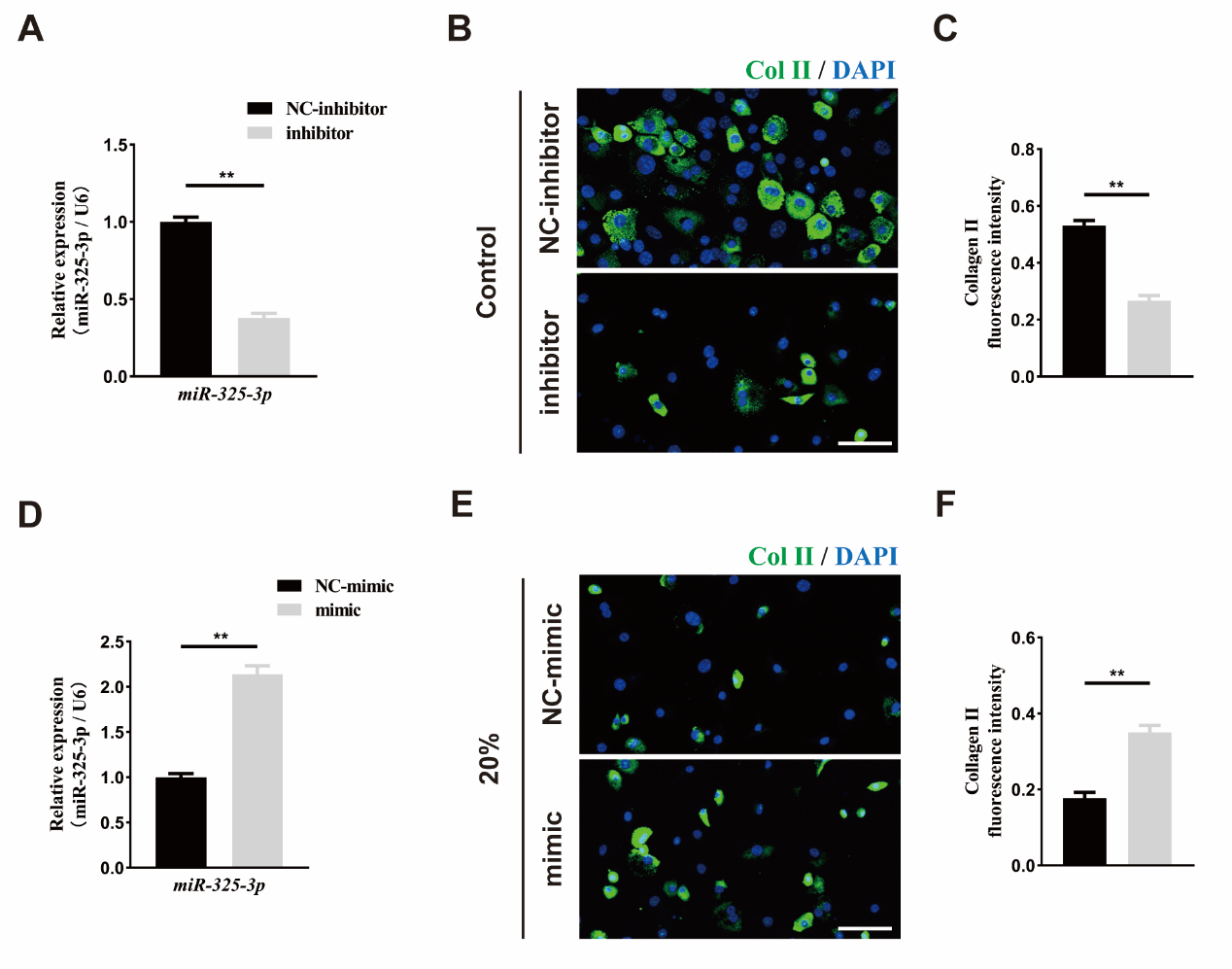


**Figure S1 The efficiency of miR-325-3p inhibitor and mimic in chondrocytes and the effect of miR-325-3p inhibitor and mimic on Collagen II in vitro.** (A) Quantitative PCR analysis of miR-325-3p in mouse chondrocytes treated with miR-325-3p inhibitor or NC-inhibitor. n = 3 per group. **(B-C)** Representative Collagen II (green) immunofluorescence staining and quantitative analysis in normal chondrocytes treated with miR-325-3p inhibitor or NC-inhibitor. n = 6 per group. Scale bar: 50 µm. **(D)** Quantitative PCR analysis of miR-325-3p in CTS-treated chondrocytes administrated with miR-325-3p mimic or NC-mimic. n = 3 per group. **(E-F)** Representative Collagen II (green) immunofluorescence staining and quantitative analysis in CTS-treated chondrocytes administrated with miR-325-3p mimic or NC-mimic. n = 6 per group. Scale bar: 50 µm. All data are shown as the mean ±standard deviation (SD). **P < 0.01.


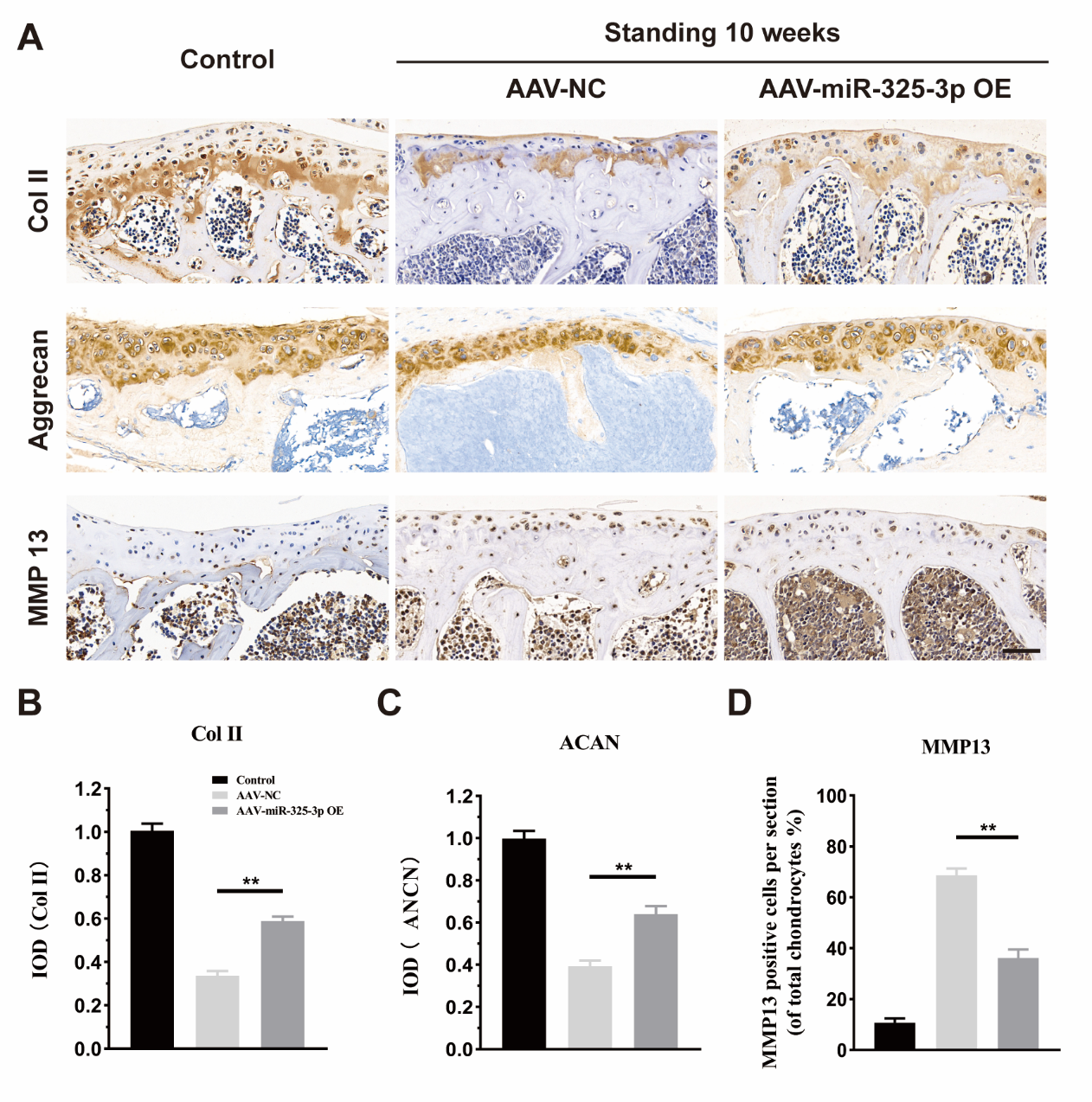


**Figure S2 AAV-miR-325-3p OE alleviates catabolism and promotes anabolism of chondrocytes in vivo. (A)** Representative immunohistochemistry images of Collagen II (top), Aggrecan (middle) and MMP13 (bottom) in LFJ cartilage in sham, AAV-NC-treated groups and AAV-miR-325-3p OE-treated groups after 10 weeks bipedal standing. **(B-D)** Quantitative analysis of Collagen II, Aggrecan and MMP13 in **(A)**. Scale bar=50 μm. n=6 per group. All data are shown as the mean ±standard deviation (SD). **P < 0.01.


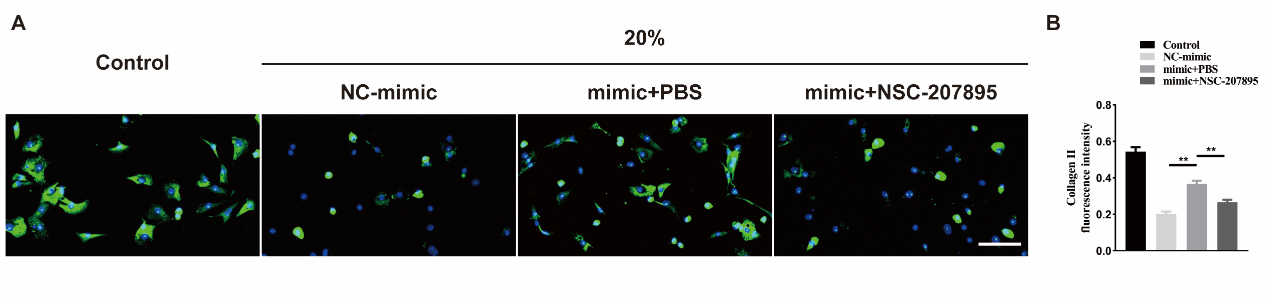


**Figure S3 The effect of NSC-207895 (p53 activator) on mechanical-stress chondrocytes after treated with miR-325-3p mimic. (A)** Representative immunofluorescence images of Collagen II in mouse chondrocytes in different groups. **(B)** Quantitative analysis of Collagen II fluorescence intensity in **(A)**. Scale bar=50 μm. n=6 per group. All data are shown as the mean ±standard deviation (SD). **P < 0.01.


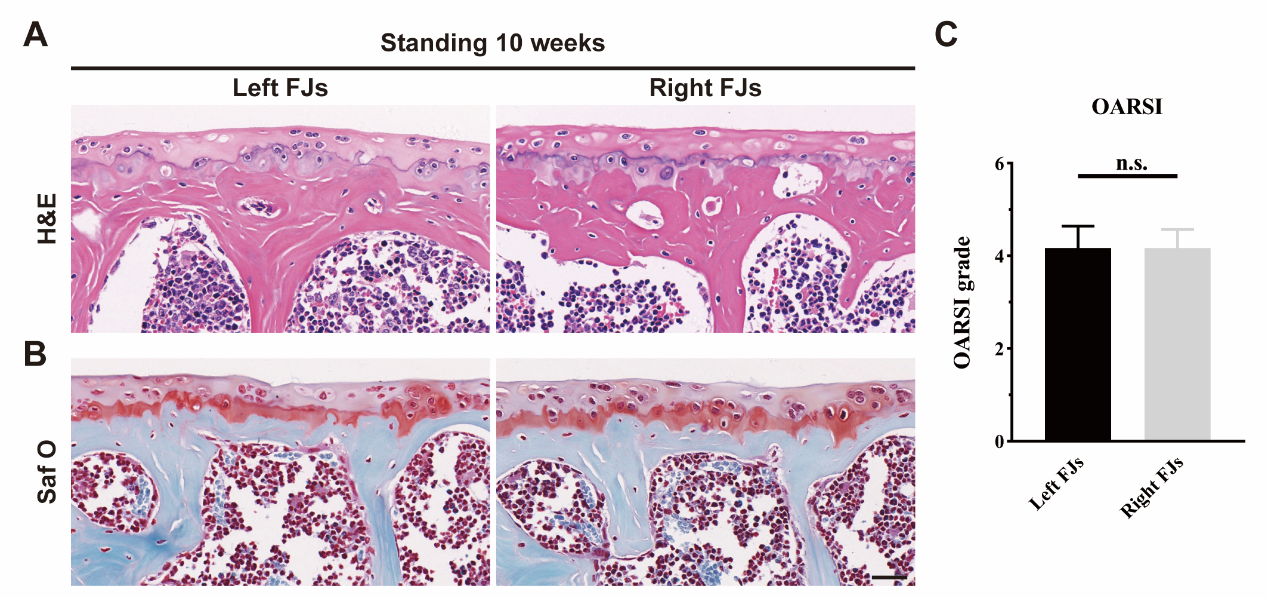


**Figure S4 Symmetrical facet joint degeneration was observed in the bipedal standing mice model. (A-B)** Representative histological images of left and right FJs (Facet Joints) with Hematoxylin-eosin (H&E) and Safranin O/Fast Green staining after 10 weeks standing in mice. **(C)** Quantitative analysis of Osteoarthritis Research Society International (OARSI) score in **(B)**; Scale bar=50 μm. All data are shown as the mean ±standard deviation (SD). n=6 per group. All data are shown as the mean ±standard deviation (SD). ‘n.s.’ represents non-significant (two-tailed Student’s t-test).
