# Supplementary material for: Mechanical overloading-induced miR-325-3p reduction promoted chondrocyte senescence and exacerbated facet joint degeneration
Source: Arthritis Res Ther. 2023 Apr 4;25:54. doi: 10.1186/s13075-023-03037-3 (PMC10071751; doi:10.1186/s13075-023-03037-3)
Supplement: Supplementary file 2 — Additional file 2: Table S1. Antibodies. Table S2. Sequences of mimic and inhibitor. Table S3. Primers for qRT-PCR. [file 13075_2023_3037_MOESM2_ESM.docx]

**Supplemental tables：**

Table S1 Antibodies

| Antibodies | Application and Dilution | Catalog numbers and species |
| --- | --- | --- |
| Collagen II | Immunofluorescence; 1:200 | ab34712; Rabbit |
| P21 | Immunofluorescence; 1:500 | ab188224; Rabbit |
| Collagen II | IHC; 1:200 | ab34712; Rabbit |
| Aggrecan | IHC in human; 1:200 | ab186414; Rabbit |
| Aggrecan | IHC in mice; 1:500 | GB11373 (Servicebio); Rabbit |
| MMP13 | IHC; 1:200 | ab39012; Rabbit |
| P21 | IHC; 1:200 | ab188224; Rabbit |
| P53 | IHC; 1:500 | ab26; Rabbit |
| P21 | Western blot; 1:1000 | Zenbio (381102; Rabbit) |
| P16 | Western blot; 1:1000 | Zenbio 380928; Rabbit |
| P53 | Western blot; 1:1000 | Wanleibio (WL01919; Rabbit) |
| Actin | Western blot; 1:50000 | Proteintech (66009-1-Ig; Mouse) |
| Goat Anti-Rabbit IgG H&L (Alexa Fluor® 594) | Immunofluorescence; 1:400 | Ab150080; Goat |
| Goat Anti-Rabbit IgG H&L (Alexa Fluor® 488) | Immunofluorescence; 1:400 | Ab150077; Goat |
| Goat anti-Mouse IgG (H+L) Secondary Antibody, HRP | Western blot; 1:5000 | #31430; Goat |
| Goat anti-Rabbit IgG (H+L) Secondary Antibody, HRP | Western blot; 1:5000 | #31460; Goat |
| Goat F(ab')2 Anti-Rabbit IgG F(ab')2 (HRP) preadsorbed | IHC; 1:1000 | ab6112; Goat |

Table S2 Sequences of mimic and inhibitor

| miR-325-3p | Forward (5’–3’) | Reverse (5’–3’) |
| --- | --- | --- |
| mimic | UUUAUUGAGCACCUCCUAUCAA | UUGAUAGGAGGUGCUCAAUAAA |
| NC-mimic | UCACAACCUCCUAGAAAGAGUAGA | UCUACUCUUUCUAGGAGGUUGUGA |
| inhibitor | - | UUGAUAGGAGGUGCUCAAUAAA |
| NC-inhibitor | - | UCUACUCUUUCUAGGAGGUUGUGA |

Table S3 Primers for qRT-PCR

| Primers | Forward (5’–3’) | |
| --- | --- | --- |
| mmu-miR-325-3p | CGCCTTTATTGAGCACCTCCTATCAA | |
| Primers | Forward (5’–3’) | Reverse (5’–3’) |
| *p16* | CGCAGGTTCTTGGTCACTGT | TGTTCACGAAAGCCAGAGCG |
| *p21* | CCTGGTGATGTCCGACCTG | CCATGAGCGCATCGCAATC |
| *p53* | GCGTAAACGCTTCGAGATGTT | TTTTTATGGCGGGAAGTAGACTG |
| *Col2a* | GGGAATGTCCTCTGCGATGAC | GAAGGGGATCTCGGGGTTG |
| *Mmp13* | CTTCTTCTTGTTGAGCTGGACTC | CTGTGGAGGTCACTGTAGACT |
| *PAI-1* | TCTGGGAAAGGGTTCACTTTACC | GACACGCCATAGGGAGAGAAG |
| *IL-6* | TAGTCCTTCCTACCCCAATTTCC | TTGGTCCTTAGCCACTCCTTC |
| *TGF-β* | TCTGCATTGCACTTATGCTGA | AAAGGGCGATCTAGTGATGGA |
| *GAPDH* | AGCAAGGACACTGAGCAAGA | GGGGTCTGGGATGGAAATTGT |
